# Concordance analysis between different methodologies used for identification of oral isolates of *Candida* species

**DOI:** 10.25100/cm.v49i3.3774

**Published:** 2018-09-30

**Authors:** Alejandra Zuluaga, Karen Arango-Bustamante, Diego H. Caceres, Zilpa A. Sánchez-Quitian, Verónica Velásquez, Beatriz L. Gómez, Claudia M. Parra-Giraldo, Natalia Maldonado, Luz E. Cano, Catalina de Bedout, Raúl E. Rivera

**Affiliations:** 1 Unidad de Micología Médica y Experimental, Corporación para Investigaciones Biológicas (CIB), Medellín, Colombia.; 2 ORISE Fellow with the Mycotic Diseases Branch, Centers for Disease Control and Prevention (CDC), Atlanta, USA.; 3 Unidad de Investigación en Proteómica y Micosis Humanas, Grupo de Enfermedades Infecciosas, Departamento de Microbiología, Facultad de Ciencias, Pontificia Universidad Javeriana, Bogotá, Colombia.; 4 Escuela de Medicina y Ciencias de la Salud, Universidad del Rosario, Bogotá, Colombia.; 5 Laboratorio Médico de Referencia S.A.S, Grupo GERMEN, Medellín, Colombia.; 6 Escuela de Microbiología, Universidad de Antioquia, Medellín, Colombia.; 7 Programa de Odontología, Universidad Antonio Nariño, Armenia - Quindío, Colombia.

**Keywords:** Candida, mouth mucosa, diagnosis, Polymerase Chain Reaction, Spectrometry, Mass, Matrix-Assisted Laser Desorption-Ionization, Colombia, Candida, mucosa bucal, diagnóstico, reacción en cadena de la polimerasa,espectrometría, masas, matrix asistida por láser de desorción-ionización, Colombia

## Abstract

**Background:**

The yeasts species determination is fundamental not only for an accurate diagnosis but also for establishing a suitable patient treatment. We performed a concordance study of five methodologies for the species identification of oral isolates of *Candida* in Colombia.

**Methods:**

Sixty-seven *Candida* isolates were tested by; API® 20C-AUX, Vitek®2 Compact, Vitek®MS, Microflex® and a molecular test (panfungal PCR and sequencing). The commercial cost and processing time of the samples was done by graphical analysis.

**Results:**

Panfungal PCR differentiated 12 species of *Candida*, Vitek®MS and Microflex® methods identified 9 species, and API® 20C-AUX and Vitek®2 Compact methods identified 8 species each. Weighted Kappa (wK) showed a high agreement between Panfungal PCR, Vitek®MS, Microflex® and API® 20C-AUX (wK 0.62-0.93). The wK that involved the Vitek®2 Compact method presented moderate or good concordances compared with the other methods (wK 0.56-0.73). Methodologies based on MALDI TOF MS required 4 minutes to generate results and the Microflex® method had the lowest selling price.

**Conclusion:**

The methods evaluated showed high concordance in their results, being higher for the molecular methods and the methodologies based on MALDI TOF. The latter are faster and cheaper, presenting as promising alternatives for the routine identification of yeast species of the genus *Candida*.

## Resumen

**Introducción:**

La clasificación a nivel de especies de las levaduras del género *Candida* de origen clínico es fundamental para el diagnóstico y la instauración de un adecuado tratamiento para el paciente. Se realizó un estudio de concordancia de cinco metodologías usadas para la identificación de aislamientos orales de *Candida* spp en Colombia.

**Métodos:**

Sesenta y siete aislamientos de *Candida* spp fueron identificados a nivel de especie utilizando; API® 20 C AUX‚ Vitek® 2 Compact, MALDI TOF (Vitek® MS y Microflex®) y una prueba molecular, PCR Panfungal y secuenciación. Un análisis del costo comercial y tiempo de procesamiento de las muestras por cada método fue realizado mediante el análisis gráfico de ambas variables.

**Resultados:**

La PCR Panfungal y secuenciación diferenció 12 especies de *Candida*‚ los métodos Vitek® MS y Microflex® identificaron 9 especies y los métodos API® 20 C AUX y Vitek® 2 Compact identificaron 8 especies. El análisis de Kappa ponderado (wK) demostró una concordancia alta entre los métodos PCR Panfungal y secuenciación‚ Vitek® MS‚ Microflex® y API® 20 C AUX‚ concordancias agrupadas en las categorías buena y muy buena (wK 0.62 - 0.93); los Kp que involucraron el método Vitek® 2 Compact presentaron concordancias moderadas o buenas frente a los otros métodos (wK 0.56 - 0.73). Las metodologías basadas en MALDI TOF MS requirieron 4 minutos para generar un resultado y el método Microflex® fue el método que en nuestro medio presentó el menor precio de venta del servicio.

**Conclusión:**

Los métodos evaluados presentaron una alta concordancia en sus resultados‚ siendo más alta para los métodos moleculares y las metodologías basadas en MALDI TOF MS; estas últimas son metodologías más rápidas, económicas y precisas, las cuales se presentan como alternativas prometedoras para la identificación rutinaria de especies de levaduras del género *Candida*.

## Introduction

The genus *Candida* consists of a group of ubiquitous yeasts with diverse characteristics. The best-known species in the group is *Candida albicans,* as it is the main species related to most yeast infections in humans, however, an increase in the number of infections caused by species different from *C. albicans* has been observed. These emerging species have become more important, since some have profiles of resistance to commonly used antifungal drugs, especially azoles and echinocandins ^1^. Based on the above, the correct identification of yeasts of the genus *Candida* is one of the greatest challenges at present, because this could delay the establishment of suitable treatment in patients with invasive fungal infections. For this reason, it is necessary to have fast and accurate tests for identification of clinical isolates ^2^.

Currently, there are several methodologies for the identification of yeasts, some using chromogenic medias such as CHROMagar™ *Candida*, that allow a presumptive identification of the most common and relevant clinical species (*C. albicans* and *C. tropicalis*). It is important to know that many authors emphasize the importance of complementing such identification with other phenotypic tests that allow species confirmation ^3^. Similarly, there are commercial systems such as API^®^ 20 C AUX or Vitek^®^ 2 Compact system for the identification of yeasts, using methodologies that are based on biochemical identification. They have the disadvantage that they can generate misidentification due to the lack of experience of the laboratory technician in the interpretation of results, and with some frequency these systems are not able to differentiate species with similar biochemical profiles, because some emergent species are not included in their databases ^4^. Mass spectrometry, based on the matrix-assisted laser desorption ionization time-of-flight (MALDI TOF) methodology, has emerged as a valuable method for the identification of microorganisms, and with good performance in the identification of yeasts, for its speed and precision ^5^ ^,^ ^6^. For these reasons, this technology has been used more frequently in clinical laboratories, the commercial systems Microflex^®^ (Bruker Daltonics GmbH, Leipzig, Germany) and Vitek^®^ MS (bioMérieux, Marcy, L'Etoile, France) being the most popular ^6^ ^,^ ^7^.

Other methodologies employ molecular techniques based on the sequencing of nucleic acids, which has been used as a reference method to make comparisons with other identification tests, because it provides more accurate identification ^8^ ^,^ ^9^. Additionally, sequencing techniques permit the identification of cryptic species such as *C. orthopsilosis*, *C. nivariensis*, or *C. bracarensis* with high precision, which frequently exhibit resistance to antifungal agents ^9^. As a limitation, the use of DNA sequencing techniques is limited to laboratories with special spaces, has equipment requirements, and requires highly trained personnel ^10^.

Considering that there are few studies that have analyzed the concordance of yeasts identification methods of, the principal aim of this study was to evaluate the concordance between five different methods, based on biochemical testing, mass spectrometry and DNA sequencing, that are used for the identification of yeasts of the genus *Candida*.

## Materials and Methods

### Population and study site

Oral rinses were obtained during the 2014 from 98 healthy adult individuals attending at the Universidad Antonio Nariño dental clinics, located in nine Colombian cities (Armenia, Bogota, Bucaramanga, Cucuta, Ibagué, Neiva, Palmira, Popayan and Villavicencio). These individuals did not have known systemic disease, although some had localized pathological processes. Samples from patients with systemic disease and from patients receiving antibiotic, antifungal or corticosteroid treatment in the last 6 months were excluded from the analyses.

### Isolates

Mouthwashes were immediately submitted to the Laboratory of the Medical and Experimental Mycology Unit, at the Corporación para Investigaciones Biológicas (CIB) in Medellín, Colombia. Samples were processed using Sabouraud Dextrosa™ agar with antibiotics (BD™, reference 210950) ^11^ and with CHROMagar™ *Candida* (CHOMagar Microbiology, Paris, France) ^12^, which allowed verification of the possibility of mixed infections by several species of Candida from the primary cultures. The cultures were incubated at 25° C for 20 days, with weekly readings to evaluate the type of growth. The recovered isolates (n= 67) were stored in sterile distilled water at 4° C and in a medium composed of skim milk (BD™, reference 232100) at -20° C.

### Identification of yeast

Identification of the isolates was performed using the following methodologies: 1) CHROMagar™ *Candida* (CHOMagar Microbiology, Paris, France). 2) API® 20 C AUX (bioMérieux, Marcy, L'Etoile, France). 3) Vitek^®^ 2 Compact automated system (bioMérieux, Inc., Hazelwood, MO, USA). 4 and 5) Mass spectrometry based on the MALDI TOF MS (matrix assisted laser desorption / ionization) technique in Vitek^®^ MS (bioMérieux, Marcy, L'Etoile, France) and Microflex^®^ (Bruker Daltonics GmbH, Leipzig, Germany). 6) Polymerase Chain Reaction (PCR) Panfungal and sequencing.

#### Methodologies used for the identification of yeasts

1. CHROMagar™ *Candida*: After surface growth on this medium, the color of each of the colonies was checked to classify them according to the following characteristics: *C. albicans/dubliniensis* complex colonies showed medium to light green color, *C. tropicalis* blue colonies, and other species presented pink or light to dark lilac color, or their natural cream color ^12^ ^,^ ^13^. 

2. API® 20 C AUX: Testing was performed following the manufacturer's recommendations (bioMérieux, Marcy-l'Etoile, France) ^14^. After the incubation period (48 h at 25° C), panels were checked visually. The numerical profile obtained for each isolate was interpreted using the Apiweb^TM^ software (bioMérieux, reference: 40 011). 

3. Vitek^®^ 2 Compact: The inoculum was prepared in 3 mL of 0.45% saline solution, using a pure culture of no more than 24 h growth. The suspension was adjusted to a McFarland turbidity range of 1.8-2.2 using the DensiCheck®. The final inoculum was automatically dispensed into the kit identification cards (YST, reference: 21343), and incubated using the Vitek^®^ 2 Compact equipment (bioMérieux, Durham, NC). The final identification was classified as follows: "excellent," "very good," "good," "acceptable," or "with low discrimination," depending on confidence level and percentage of discrimination for each identification provided by the equipment’s software ^15^. 

4. MALDI TOF MS: This was done using two commercial platforms; the Vitek® MS equipment (bioMérieux, Marcy, L'Etoile, France) and the Microflex^®^ (Bruker Daltonics GmbH, Leipzig, Germany) Biotype Library 3.0. The methodological details corresponding to each commercial method are described below. 

4.1 Vitek^®^ MS: A single pure colony (growth 24-72 h) was deposited in a single well of the Vitek^®^ MS plate. Cells were lysed with 0.5 μL of 25% concentration formic acid (Ref: 411072) and allowed to dry at room temperature (1-2 min). After drying, 1 μL of the CHCA matrix (bioMérieux, Marcy, L'Etoile, France, reference: 411071) was added. Tests were performed after the final mixture was completely dry. The peak spectrum was analyzed using the MS-ID server (MS-ID [CE / IVD] database) ^16^. 

4.2 Microflex^®^: Isolates grown for no more than 24 h at 37( C were tested by direct extraction methodology in plate or by extraction with formic acid. For direct extraction, a single colony was deposited directly in the MALDI TOF plate, allowed to dry at room temperature; after dry, 1 μL of 100% concentration formic acid was added, and then was covered with 1 μl of HCAA matrix (α-cyano-4 hydroxycinnamic acid - HCCA). After drying for a second time at room temperature, the treated samples were analyzed using the Biotyper machine and the final mass spectra were analyzed using the FlexControl software (version 3.0) and the MALDI Biotyper RTC. Strains that had a low score in the direct test identification or in which the identification was not possible were extracted with formic acid, following the manufacturer's instructions (Bruker Daltonik GmbH, Bremen, Germany) ^17^. Each sample was served in duplicate to verify the reproducibility. 

5. Panfungal PCR and sequencing: The D1/D2 region of the 28S rRNA gene was amplified, following the international guidelines for molecular identification of fungi ^18^. The ITS 1-4 (Internal Transcribed Spacer) region was also amplified for the identification of cryptic species in *Candida parapsilosis* and *Candida glabrata* isolates^13^. Genomic DNA was extracted from isolated colonies grown on Sabouraud agar using the QIAamp DNA mini kit (QIAGEN, Germantown, MD), following the manufacturer's recommendations. Molecular markers were amplified using primers and protocols previously described for the D1/D2 and ITS 1-4 regions ^19^ ^,^ ^20^. Amplified products from the D1/D2 region (~ 600 bp) and the ITS region 1-4 (600-900 bp) were shipped to Macrogen laboratories (Maryland, USA) for sequencing. Editing and aligning of sequences were performed using the Sequencher 5.0 software (Gene Code Corporation). A search was made to establish similarity/homology in two databases: the NCBI (BLAST) (National Biotechnology Information Center, Washington, DC) and the CBS-KNAW (Fungal Diversity Center). 

### Methodological design and statistical analysis

Variables analyzed in this study were summarized by calculating both absolute and relative frequencies. A concordance analysis was performed to evaluate the agreement between different methods used for yeast identification. The Weighted Kappa (wK) values and their respective 95% confidence intervals (95% CI) were calculated. The concordance analysis was performed in two ways, as follows: The first analysis was done by regrouping the results obtained by the API^®^ 20 C AUX, Vitek^®^ 2 Compact, Vitek^®^ MS, Microflex^®^ and panfungal PCR and sequencing, in the following three categories of results: *C. albicans/dubliniensis*, *C. tropicalis*, and *Candida* spp different than *Candida albicans/dubliniensis/tropicalis.* This regrouped result was done to compare the five previous mentioned methodologies against the CHROMagar™ *Candida*. The second analysis compared the results of the API^®^ 20 C AUX, Vitek^®^ 2 Compact, Vitek^®^ MS, Microflex^®^ and panfungal PCR and sequencing, and in this second analysis the panfungal PCR and sequencing method was selected as the reference methodology. In addition, a cost/turnaround time results analysis was performed, using the sale price of the service and the turnaround time (excluding the times of the pre-analytical and post-analytical phases) from local laboratory providers in the city of Medellín, Colombia. Information was collected through telephone calls or email. Interpretation was performed using graphic analysis of the two variables (cost and turnaround time). Statistical analyses were performed using the statistical package STATA 8.0^®^ and graphics using Microsoft Excel 2010^®^ software.

### Ethics

The protocol was approved by the ethical committees of the Universidad Antonio Nariño, Armenia - Quindío, Colombia.

## Results

Sixty-seven *Candida* isolates were recovered from 98 oral rinses (68% positivity). In the initial analysis using the CHROMagar™ *Candida* the 67 isolates were classified as follows: 39 (58%) isolates as *C. albicans/dubliniensis*, 4 (6%) as *C. tropicalis* and 24 (36%) isolates such as *Candida* different than *Candida albicans/dubliniensis/tropicalis*. The first concordance analysis to compare the CHROMagar™ *Candida* against the other five methodologies (regrouped results) gave the following Weighted Kappa (wK) results: vs API^®^ 20 C AUX = 1.00 (95% CI= 1.00-1.00); vs Vitek^®^ 2 Compact = 0.87 (95% CI= 0.75-0.96); vs Vitek® MS= 0.92 (95% CI= 0.80-0.99); vs Microflex® = 0.97 (95% CI= 0.94-1.00); and vs panfungal PCR and sequencing = 0.98 (95% CI= 0.95-1.00).

**Figure 1:**
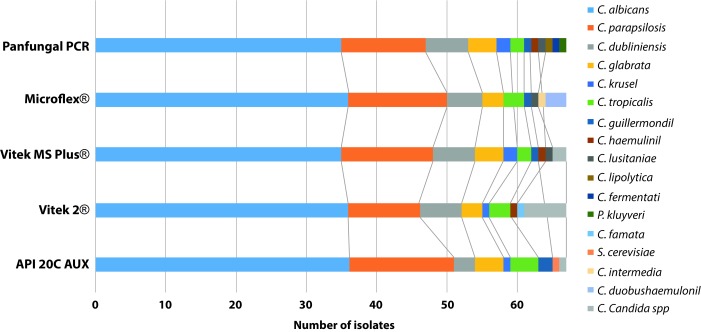
Distribution of species classified according to the identification methodology.

In the results of yeast identification, excluding the CHROMagar™ *Candida* results, it was observed that Panfungal PCR and sequencing was the method that identified the largest number of species (n= 12), following by Mass spectrometry methods, Vitek® MS and Microflex®, which identified 9 different *Candida* species each, and finally the biochemical methods API® 20 C AUX and Vitek® 2 Compact, which identified 8 different *Candida* species each. The description of the species identified by each methodology is summarized in Figure 1.

**Figure 2:**
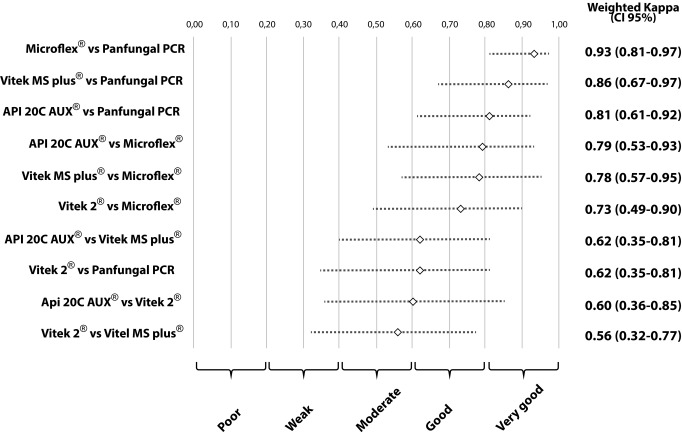
Concordance analysis of five methodologies for the identification of oral isolates of *Candida* species.

The second concordance study showed a high concordance between the results obtained with Panfungal PCR and sequencing, Vitek® MS, Microflex® and API® 20 C AUX, these results were grouped into the good and very good categories. The wK that involved the Vitek® 2 Compact method were characterized by moderate or good results compared with the other methods (Panfungal PCR and sequencing, Vitek® MS, Microflex® and API® 20 C AUX). A detailed analysis of the concordances and discordances comparing all the methods is given in Table 1. Values of wK observed and their respective 95% CI are summarized in Figure 2.

**Table 1:** Comparison of concordances and discrepancies of the API^®^ 20 C AUX, Vitek^®^ 2 Compact, Vitek^®^ MS and Microflex^®^ against the reference method Panfungal PCR and sequencing.

	Reference method	API® 20 C AUX		Vitek^**®**^ 2 Compact		Vitek^**®**^ MS		Microflex^**®**^	
* *	Panfungal PCR and sequencing (n)	C (n)	D (n)	C (n)	D (n)	C (n)	D (n)	C (n)	D (n)
*C. albicans*	35	33	2	34	1	33	2	35	0
*C. parapsilosis*	12	12	0	10	2	12	0	12	0
*C. dubliniensis*	6	2	4	6	0	5	1	5	1
*C. glabrata*	4	4	0	2	2	4	0	3	1
*C. tropicalis*	2	2	0	2	0	2	0	2	0
*C. intermedia*	2	0	2	0	2	2	0	1	1
*C. guilliermondii*	1	1	0	0	1	1	0	1	0
*C. lusitaniae*	1	0	1	1	0	1	0	1	0
*C. haemulonii*	1	0	1	1	0	1	0	1	0
*C. lipolytica*	1	0	1	0	1	0	1	0	1
*C. fermentati*	1	0	1	0	1	0	1	0	1
*Pichia kluyveri*	1	0	1	0	1	0	1	0	1
**Total**	67	54	13	56	11	61	6	61	6

n: número

C: concordances,

D: discrepancies

To confirm that the previous methods did not fail to detect species of the complex *Candida parapsilosis* and *Candida glabrata*, a PCR and sequencing analysis of the ITS 1-4 region was performed, determining no presence of cryptic species.

**Figure 3:**
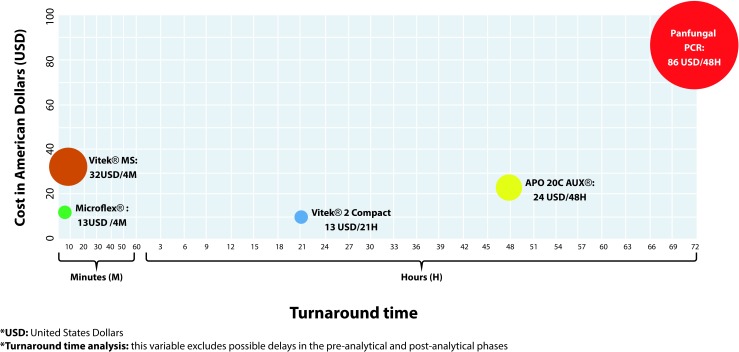
Results of the cost/turnaround time analysis.

The analysis of costs/turnaround time results showed that the methods based on the MALDI TOF technology (Microflex® and Vitek® MS) required less time to generate a result (4 minutes), and in addition, the Microflex® method had the lower commercial cost (13 US Dollars). The panfungal PCR and sequencing was the method that required most time to generate a final report (3 days) and had the highest commercial value (86 USD). The analysis that compares the costs and turnaround time results for the tests evaluated in this report is summarized in Figure 3.

## Discussion

This study evaluated five different methodologies for the identification of clinical isolates of the *Candida* genus obtained from oral rinses. In general, we observed good concordance between the CHROMagar™ *Candida* and the other five methodologies evaluated in this study. The Vitek® 2 Compact method had the lowest agreement and the highest agreement was observed with the API® 20 C AUX. It is important to mention that use of CHROMagar™ *Candida* allows for identification of the presence of multiple species in the same clinical sample and gives a presumptive identification of the associated *Candida* species ^21^ ^,^ ^22^.

Most of the five methods analyzed showed high concordances grouped into good and very good categories. Previous studies have shown that phenotypic methods, based on biochemical tests such as the API® 20 C AUX and the Vitek® 2 Compact system, are the most used in clinical laboratories ^23^ ^,^ ^24^. In this study, it was observed that these two methods had the lowest capacity to differentiate *Candida* species and also presented lower values of wK, especially when comparing the Vitek® 2 Compact method with the other methodologies. Other authors have reported that these methods may present discrepancies in identifying less common *Candida* species and especially those that are closely related ^23^ ^,^ ^25^. In addition, the accuracy of these methods is greatly reduced if the laboratory technician does not perform the additional tests required for the identification. 

In our study, Panfungal PCR and sequencing was able to identify species such as *Candida fermentati* and *Candida haemulonii*, which are emerging species, recently classified as species complex, that are difficult to identify by conventional methods and whose identification can become a challenge, because these species have not been included in the databases of most of the different identification systems available ^26^ ^,^ ^27^. In addition, some of these emerging species have shown a decrease in sensitivity to antifungal agents, and consequently, this increases the difficulty in the management of patients with invasive *Candida* infections ^26^ ^-^ ^28^. In our study, none of the methodologies analyzed, except for Panfungal PCR and sequencing, had the ability to identify *Candida lipolytica* (n= 1), *Candida fermentati* (n= 1) and *Pichia kluyveri* (n= 1), species that have been reported mainly for industrial use, but, also have the capacity to behave as pathogens in humans according to host conditions ^26^ ^,^ ^29^ ^-^ ^32^.

The methodologies based on MALDI TOF (Microflex® and Vitek® MS) presented high concordance compared with the reference method, panfungal PCR and sequencing. The analysis of concordance between these two technologies showed a very good agreement, due to the fact that both systems identified the same number of species, but not all of them corresponded to the same species, since there were inconsistencies in the identifications in 6 of the 67 analyzed isolates. There are several platforms of MALDI TOF, and differences will depend on the libraries of mass spectra and the algorithms of identification that each platform has ^33^ ^,^ ^34^. In this study the Microflex® was able to identify all the isolates at species level. When using the Vitek® MS spectrometer, three major discordances were observed (this platform was not able to determine the final identification at species level in three isolates), when compared with the reference method, affecting its wK value. Many studies have shown that these techniques are faster and more accurate in the identification of yeasts from crops, which has a high relevance in terms of time and costs ^7^ ^,^ ^35^ ^,^ ^36^.

The panfungal PCR and sequencing advantage is that this technique allows the identification of cryptic species or complex species, but it is recommended to combine the amplification of the D1/D2 region with other targets in the ITS region ^24^ ^,^ ^26^. In this report, although the presence of cryptic species of *Candida parapsilosis* and *Candida glabrata* were not observed, it is important to use methodologies that are able to detect them, mainly due to the emergence of species that are difficult to manage ^9^ ^,^ ^37^ ^,^ ^38^. In addition, this technique would be the best option, despite its cost, for those identifications that are doubtful or where other methodologies have low discriminatory power.

When we analyzed the costs and turnaround time results, we could determine that the methods based on the MALDI TOF technology (Microflex® and Vitek® MS) were the ones that offered the least time for results, as previously reported, and when comparing this methodology with other conventional methods, our results also reflected a reasonable and competitive cost ^39^. It should be noted that this technology allows precise results, surpassing the conventional identification techniques ^40^ ^,^ ^41^. This technology has shown great potential for fast and accurate identification, and this finding demonstrates the need to make its methodologies more available.

It is important to mention that delays can occur in the generation of results, as a result of factors that are not inherent to the methodology or external, such as the day that the isolation arrives in the laboratory, the viability of the isolate, the need for additional extraction steps, the days designated in the laboratory for sample processing, the availability of laboratory personnel, or turnaround time for sequencing analysis. Changes in some of these factors can make a significant difference when delivering a timely outcome and it would help to implement changes at the laboratory level to improve the different processes.

The present study was limited to the evaluation of *Candida* species isolated from oral mucosa. Although it was possible to show a wide variety of species identified between the different methods, it would be pertinent for future investigations to carry out the same evaluation in other clinical specimens. In addition, the cost and time analysis was only carried out in the city of Medellín, Colombia. In Colombia the number of laboratories that offer molecular methods or MALDI TOF technology is limited, which may increase the costs of these tests in a significant way, mainly due to the exclusivity of the service.

## Conclusion

It is necessary to implement identification methods that facilitate access to fast and reliable results, but at the same time, help to optimize the economic resources once those are implemented in the daily routine. In this work, it was shown that the systems based on the MALDI TOF technology, despite being methodologies that initially required a substantial economic investment, proved to be fast, economical (commercial value of the test) and precise, which are presented as promising alternatives for the routine identification of yeasts of the *Candida* genus. For those laboratories not able to access molecular or mass spectrometry tests, the use of automated tests could be a good alternative if the laboratorian technician follows the specification provided by the manufacture, and pays special attention to those results that involve uncommon species or discrepant characteristics presented in the isolate (morphology, susceptibility patter and other additional taxonomy keys).
