# Supplementary material for: Concordance analysis between different methodologies used for identification of oral isolates of Candida species
Source: Colomb Med (Cali). 2018 Sep 30;49(3):193–200. doi: 10.25100/cm.v49i3.3774 (PMC6220485; doi:10.25100/cm.v49i3.3774)
Supplement: Related texts

# Research article: Análisis de concordancia de diferentes metodologías para la identificación de aislamientos orales de especies de *Candida*

## Introducción

Los hongos del género *Candida* son un grupo de levaduras ubicuas y con características muy diversas. Una de las especies más conocida es *Candida albicans* ya que es la principal especie relacionada en la mayoría de infecciones causadas por levaduras, sin embargo, actualmente se ha observado un incremento de las infecciones causadas por especies diferentes, anteriormente menos comunes, pero que hoy en día han adquirido mayor importancia, ya que algunas presentan perfiles de sensibilidad disminuida y resistencia a los antimicóticos de uso habitual, en especial a los azoles y equinocandinas ^1^. Con base en lo anterior, la correcta identificación de levaduras del género *Candida* es uno de los retos más grandes existentes en la actualidad, en especial, porque esto pudiera retrasar la instauración de un adecuado tratamiento en el paciente, particularmente, en el manejo de las infecciones fúngicas invasoras. Para esto, se hace necesario contar con pruebas que sean rápidas y precisas para la identificación oportuna de levaduras de interés clínico ^2^. 

Actualmente, existen diversas metodologías para la identificación de levaduras, algunas emplean medios cromogénicos como el CHROMagar^TM^ *Candida* que permiten tener una identificación presuntiva de una manera rápida, logrando identificar especies de importancia clínica como *C. albicans*, *C. tropicalis* y *C. krusei*, sin embargo, muchos autores resaltan la importancia de complementar esta identificación con otras pruebas fenotípicas que permitan la confirmación a especie ^3^. De igual manera, existen sistemas comerciales como el API® 20 C AUX o el sistema Vitek® 2 Compact utilizados para la identificación de estas levaduras, los cuales están basados en pruebas bioquímicas. Tienen la desventaja que pueden proporcionar identificaciones erróneas por la falta de experiencia del laboratorista al interpretar los resultados y, con alguna frecuencia estos sistemas no son capaces de diferenciar especies con perfiles bioquímicos similares y existe la limitante de la inclusión oportuna de especies emergentes en sus bases de datos ^4^. La espectrometría de masas, basada en la metodología MALDI TOF (de sus siglas del inglés: *matrix-assisted laser desorption ionization time-of-flight*), ha surgido como un método valioso para la identificación de microorganismos en general, y con un buen desempeño para la identificación de levaduras, por su rapidez, precisión y bajo precio de venta de servicio ^5^^,^^6^. Por tales motivos, esta tecnología ha comenzado a emplearse con mayor frecuencia en nuestro medio, siendo los sistemas comerciales Microflex® (Bruker Daltonics GMBH, Leipzig, Alemania) y Vitek® MS (bioMérieux, Marcy, L’Etoile, Francia) los más populares ^6^^,^^7^. 

Otras de las metodologías utilizadas son las técnicas moleculares basadas en la secuenciación de ácidos nucléicos, las cuales han sido empleadas como método de referencia para hacer comparaciones frente a otras pruebas de identificación debido a que proporcionan una identificación más precisa ^8^^,^^9^. Adicionalmente, las técnicas de secuenciación permiten identificar especies crípticas como *C. metapsilosis*, *C. orthopsilosis*, *C. nivariensis*, *C. bracarensis*, que exhiben frecuentemente resistencia a los antifúngicos que comúnmente son empleados contra estos patógenos ^9^. La PCR panfungal y secuenciación tiene como limitante la complejidad para ser implementadas en un laboratorio ya que requiere espacios físicos, equipamiento especial y personal altamente entrenado ^10^. 

Con todo lo expuesto anteriormente, y teniendo en cuenta que son escasos los estudios que analizan la concordancia de los métodos empleados para la identificación de levaduras y la importancia médica que tiene conocer los agentes causales de las diferentes infecciones causadas por *Candida* spp, el objetivo de este estudio fue evaluar la concordancia entre cinco diferentes métodos, tanto convencionales como moleculares y de espectrometría de masas, empleados para la identificación de levaduras del género *Candida.*

## Materiales y Métodos 

### Población y sitio de estudio

Se incluyeron muestras de enjuague bucal que se obtuvieron a partir de 98 individuos adultos sanos durante el año 2014 que asistieron a las clínicas odontológicas de la Universidad Antonio Nariño, ubicadas en 9 ciudades colombianas (Armenia, Bogotá, Bucaramanga, Cúcuta, Ibagué, Neiva, Palmira, Popayán y Villavicencio). Estos individuos no tenían enfermedad sistémica conocida aunque, algunos tenían procesos patológicos localizados que no produjeron alteración sistémica.

Las muestras provenientes de pacientes con enfermedad sistémica y de aquellos que estuvieron recibiendo tratamiento farmacológico con antibióticos, antifúngicos o corticoesteroides en los últimos 6 meses se excluyeron de los análisis.

### Aislamientos

Los enjuagues bucales se remitieron de manera inmediata al Laboratorio de la Unidad de Micología Médica y Experimental de la Corporación para Investigaciones Biológicas (CIB) en Medellín, Colombia. Las muestras se sembraron en agar Sabouraud Dextrosa™ con antibióticos (BD™, referencia 210950) ^11^ y en medio CHROMagar^TM^ *Candida* (CHOMagar Microbiology, Paris, Francia) ^12^, lo que permitió verificar desde los cultivos primarios, la posibilidad de tener infecciones mixtas por varias especies de *Candida*. Los cultivos fueron incubados a 25° C por 20 días, con lecturas semanales para evaluar la positividad de los mismos. Los aislamientos recuperados (n= 67) fueron almacenados en agua destilada estéril a 4° C y en medio de congelación en leche “Skim milk” (BD™, referencia 232100) a -20° C. 

### Identificación de las levaduras

La identificación del género y la especie de los aislamientos fue realizada utilizando las siguientes metodologías: 1) CHROMagar^TM^ *Candida* (CHOMagar Microbiology, París, Francia). 2) API® 20 C AUX (bioMérieux, Marcy, L’Etoile, Francia). 3) Sistema automatizado Vitek® 2 Compact (bioMérieux, Inc., Hazelwood, MO, USA). 4) y 5) Espectrometría de masas basada en la técnica de MALDI TOF MS (desorción/ionización láser asistida por matriz) en los equipos Vitek® MS (bioMérieux, Marcy, L’Etoile, Francia) y Microflex® (Bruker Daltonics GMBH, Leipzig, Alemania). 6) Reacción en Cadena de Polimerasa (PCR) Panfungal y secuenciación.

#### Metodologías empleadas para la identificación de las levaduras

1. CHROMagar^TM^ *Candida*: Después del crecimiento en este medio se verificó el color de cada una de las colonias para clasificarlas de acuerdo a las siguientes características: las colonias del complejo *C. albicans/dubliniensis* presentaron un color de verde claro a mediano, las de *C. tropicalis*, de azul verdoso a azul metálico y las demás especies presentaron un color de rosado pálido o lila claro a oscuro, o bien su color crema natural en este medio ^12^^,^^13^.

2. API® 20 C AUX: La prueba se realizó siguiendo las recomendaciones del fabricante (bioMérieux, Marcy, L’Etoile, Francia) ^14^. Despúés del período de incubación (48 horas a 25° C) los paneles se inspeccionaron visualmente. El perfil numérico obtenido para cada aislamiento se interpretó utilizando el software Api*web* ^TM^ (bioMérieux, referencia: 40 011).

3. Vitek® 2 Compact: el inóculo de trabajo fue preparado en 3 mL de solución salina al 0,45% a partir de un cultivo puro de no más de 24 h de crecimiento. La suspensión se ajustó a una escala de turbidez de McFarland entre 1,8 - 2,2, empleando el equipo DensiCheck®. El inóculo final fue dispensado automáticamente en las tarjetas de identificación del kit (YST, referencia: 21343), y se incubó utilizando el equipo Vitek® 2 Compact (bioMérieux, Durham, NC). La identificación final fue clasificada de la siguiente manera: “excelente”, “muy bueno”, “bueno”, “aceptable” o “con baja discriminación”, según el nivel de confianza y el porcentaje de discriminación para cada identificación proporcionada por el software del equipo ^15^. 

4. MALDI TOF: Se realizó utilizando dos plataformas comerciales en los equipos Vitek® MS (bioMérieux, Marcy, L’Etoile, Francia) y Microflex® (Bruker Daltonics GMBH, Leipzig, Alemania). Los detalles metodológicos de cada método comercial se describen a continuación: 

4.1 Vitek® MS*:* Una sola colonia pura (crecimiento entre 24-72 h) se depositó en un solo pocillo de la placa del Vitek® MS. Las células se lisaron con 0,5 μL de ácido fórmico al 25% (Referencia: 411072) y se dejarón secar a temperatura ambiente (1-2 min). Después del secado, se adicionó 1 μL de la matriz CHCA (bioMérieux, Marcy, L’Etoile, Francia, Referencia: 411071). Las pruebas se realizaron después de que la mezcla final estaba completamente seca. El listado de picos resultantes de la adquisición se analizó utilizando el servidor MS-ID, en el que se compararon los picos de la muestra con los espectros contenidos en la base de datos MS-ID (CE/IVD) incluido en Myla^TM (^^16^. 

4.2 Microflex®: Partiendo de cultivos con no más de 24 h de crecimiento a 37º C, se realizó la identificación mediante la metodología de transferencia con extracción directa extendida en placa o mediante extracción con ácido fórmico. Para la extracción directa, se tomó una sola colonia y se realizó una capa muy delgada del microorganismo en la placa de acero MALDI, se dejó secar a temperatura ambiente, se adicionó 1 μL de ácido fórmico al 100% y se cubrió con 1 μL de matriz HCAA (α-cyano-4 hydroxycinnamic acid - HCCA) dejando secar a temperatura ambiente. Para aquellas cepas que presentaron un *score* bajo en la identificación, o en las cuales no fue posible una identificación, se les realizó extracción con ácido fórmico, la cual se hizo siguiendo las instrucciones del fabricante (Bruker Daltonik GmbH, Bremen, Alemania) con algunas modificaciones. Cada muestra fue servida por duplicado con el fin de verificar la reproducibilidad. Los perfiles fueron visualizados con el software FlexControl (versión 3.0) y el MALDI Biotyper RTC ^17^. 

5. PCR Panfungal y secuenciación: La región D1/D2 de la subunidad del complejo de genes rRNA 28S se amplificó siguiendo directrices internacionales para la identificación molecular de hongos ^18^. También se amplificó la región ITS 1-4 (Internal Transcribed Spacer) para la identificación de especies crípticas en aislamientos de *Candida parapsilosis* y *Candida glabrata* ^13^. El DNA genómico se extrajo de colonias aisladas cultivadas en agar Sabouraud usando el mini kit QIAamp DNA (QIAGEN, Germantown, MD), siguiendo las recomendaciones del fabricante. Los marcadores moleculares fueron amplificados utilizando los cebadores y protocolos previamente descritos para las regiones D1/D2 y la ITS 1-4 ^(19, 20)^.

Los productos amplificados de la región D1/D2 (~ 600 pb) y de la región ITS 1-4 (600 - 900 pb) fueron enviados a Macrogen (Maryland, EE.UU.) para la secuenciación bidireccional de Sanger. El software Sequencher 5.0 (Gene Code Corporation) se usó para editar y alinear las secuencias. Se realizó una búsqueda para establecer similitud/homología en dos bases de datos: el NCBI (BLAST) (Centro Nacional de Información de Biotecnología, Washington, DC) y el CBS-KNAW (Centro de Diversidad Fúngica).

### Diseño metodológico y análisis estadístico

Las variables analizadas en este trabajo fueron resumidas mediante el cálculo de frecuencias absolutas y relativas. Los diferentes métodos para la identificación de levaduras evaluados fueron comparados mediante un análisis de concordancia, con el cálculo del índice de Kappa ponderado (Kp) y sus respectivos intervalos de confianza del 95% (IC 95%). El análisis de concordancia fue realizado de dos formas: la primera fue reagrupando los resultados de los métodos API® 20 C AUX‚ Vitek® 2 Compact‚ Vitek® MS y Microflex® y PCR Panfungal y secuenciación en las siguientes tres categorías de resultados: *C. albicans/dubliniensis*, *C. tropicalis* y *Candida* diferente a *Candida albicans/dubliniensis/tropicalis*, lo anterior se hizo con el objetivo de comparar las metodologías de identificación con el método de tamizaje CHROMagar^TM^ *Candida*. El segundo análisis comparó los resultados finales de los métodos API® 20 C AUX‚ Vitek® 2 Compact‚ Vitek® MS y Microflex® y PCR panfungal y secuenciación, donde se tomó como método de referencia la PCR panfungal y secuenciación. Adicionalmente, fue realizado un análisis local de costos y tiempos necesario para el reporte de resultados, en el cual se analizaron las variables de precio de venta del servicio al público y el tiempo de procesamiento de la prueba para la entrega de resultados (excluyendo los tiempos de la fase pre-analítica y pos-analítica), la información analizada fue recolectada mediante contacto telefónico o vía correo electrónico con las diferentes instituciones que realizan alguna de estas metodologías en la ciudad de Medellín, Colombia. La interpretación se realizó mediante el análisis gráfico de las dos variables (costos y tiempo). Los análisis estadísticos fueron realizados utilizando el paquete estadístico STATA 8.0® y los gráficos mediante el Software Microsoft Excel 2010®.

### Responsabilidades éticas

El protocolo fue aprobado por los comités de ética de la Universidad Antonio Nariño, Armenia-Quindío, Colombia. 

## Resultados

Sesenta y siete aislamientos del género *Candida spp* recuperados a partir de 98 enjuagues bucales fueron analizadas en el laboratorio (68% de positividad). Al realizar el análisis inicial utilizando el medio CHROMagar^TM^ *Candida*, los 67 aislamientos se clasificaron como: 39 (58%) aislamientos de *C. albicans/dubliniensis*, 4 (6%) aislamientos de *C. tropicalis* y 24 (36%) aislamientos como *Candida* diferente a *Candida albicans*/*dubliniensis*/*tropicalis*. El primer análisis de concordancia comparando el CHROMagar^TM^ *Candida* con las otras cinco metodologías (resultados reagrupados), arrojaron los siguientes resultados del Kappa ponderado (Kp): vs API® 20 C AUX = 1.00 (IC 95% = 1.00 - 1.00); vs Vitek® 2 Compact = 0.87 (IC 95% = 0.75 - 0.96); vs Vitek® MS = 0.92 (IC 95% = 0.80 - 0.99); vs Microflex® = 0.97 (IC 95% = 0.94 - 1.00) y vs la PCR panfungal y secuenciación = 0.98 (IC 95% = 0.95 - 1.00).

Al analizar los resultados teniendo en cuenta cada una de las metodologías utilizadas para la identificación de levaduras (excepto el CHROMagar^TM^ *Candida*), se observó que la PCR panfungal y secuenciación, fue el método que logró diferenciar el mayor número de especies (n=12). Los métodos basados en espectometria de masas, Vitek® MS y Microflex®, identificaron nueve especies diferentes de *Candida* y finalmente, los métodos bioquímicos API® 20 C AUX y Vitek® 2 Compact, lograron identificar ocho especies diferentes de *Candida* cada uno. La descripción de las especies identificadas por metodología, se resumen en la Figura 1.

**Figura 1:** Distribución de las especies de levaduras clasificadas según el método de identificación

El segundo estudio de concordancia mostró una concordancia alta entre los resultados obtenidos con los métodos PCR panfungal y secuenciación, Vitek® MS, Microflex® y API® 20 C AUX, concordancias agrupadas en las categorías buena y muy buena. Los Kp que involucraron el método Vitek® 2 Compact, presentaron concordancias moderadas o buenas frente a los otros métodos (PCR panfungal y secuenciación, Vitek® MS, Microflex® y API® 20 C AUX). El análisis de las concordancias y discordancias comparando todos los métodos se encuentran resumidas en la Tabla 1. Los valores de Kp observados y sus respectivos IC del 95%, se encuentran resumidos en la Figura 2. 

**Tabla 1:** Comparación de concordancias y discordancias de los métodos API® 20 C AUX, Vitek® 2 Compact, Vitek® MS y Microflex® con el método de referencia PCR panfungal y secuenciación.

	Método de referencia	API® 20 C AUX		Vitek^**®**^ 2 Compact		Vitek^**®**^ MS		Microflex^**®**^	
* *	PCR panfungal y secuenciación (n)	C (n)	D (n)	C (n)	D (n)	C (n)	D (n)	C (n)	D (n)
*C. albicans*	35	33	2	34	1	33	2	35	0
*C. parapsilosis*	12	12	0	10	2	12	0	12	0
*C. dubliniensis*	6	2	4	6	0	5	1	5	1
*C. glabrata*	4	4	0	2	2	4	0	3	1
*C. tropicalis*	2	2	0	2	0	2	0	2	0
*C. intermedia*	2	0	2	0	2	2	0	1	1
*C. guilliermondii*	1	1	0	0	1	1	0	1	0
*C. lusitaniae*	1	0	1	1	0	1	0	1	0
*C. haemulonii*	1	0	1	1	0	1	0	1	0
*C. lipolytica*	1	0	1	0	1	0	1	0	1
*C. fermentati*	1	0	1	0	1	0	1	0	1
*Pichia kluyveri*	1	0	1	0	1	0	1	0	1
**Total**	67	54	13	56	11	61	6	61	6

n: número

C: concordancias,

D: discrepancias

**Figura 2:** Análisis de concordancia de cinco metodologías para la identificación de aislamientos orales de especies de *Candida*.

Para corroborar que los métodos anteriores no dejaron de detectar especies de los complejos *Candida parapsilosis* y *Candida glabrata*, se complementó con una PCR y secuenciación de la región ITS 1-4, determinando que, en los complejos de *Candida parapsilosis* (n=12) y *Candida glabrata* (n=4), no se identificaron especies crípticas. 

El análisis de costos y tiempo de procesamiento de la pruebas para generar resultados, demostró que los métodos basados en la tecnología MALDI TOF (Microflex® y Vitek® MS) requirieron menos tiempo para la generación de resultados (4 minutos), además, el método Microflex® presentó menor valor comercial (13 Dólares Americanos). La PCR panfungal y secuenciación, fue el método que requirió más tiempo para la entrega de resultados (3 días) y presentó el mayor valor comercial (86 USD). El análisis que compara las metodologías diagnósticas con base en los costos comerciales y el tiempo de procesamiento hasta la obtención de resultados, se resume en la Figura 3. 

**Figura 3:** A Resultados del análisis del costo/tiempo de respuesta. USD: Dólares Americanos. Análisis del tiempo de respuesta: esta variable excluye retrasos eventuales dentro de la fase pre-analítica y pos-analítica.

## Discusión

Este estudio evaluó cinco metodologías diferentes para la identificación de aislamientos clínicos del género *Candida* obtenidos de enjuagues bucales. En general, observamos una buena concordancia entre CHROMagar ™ *Candida* y las otras cinco metodologías evaluadas en este estudio. El método Vitek® 2 Compact tuvo la concordancia más baja y la mayor fue observada con el API® 20 C AUX. Es importante mencionar que el uso de CHROMagar ™ *Candida* permite la identificación de la presencia de múltiples especies en la misma muestra clínica y proporciona una identificación presuntiva de las especies de *Candida* asociadas ^21^^,^^22^.

La mayoría de los cinco métodos analizados demostraron concordancias altas agrupadas en las categorías buena y muy buena. Estudios previos han demostrado que los métodos fenotípicos, basados en pruebas bioquímicas como el API® 20 C AUX y el sistema Vitek® 2 Compact, son los más empleados en los laboratorios clínicos ^23^^,^^24^. En este trabajo se observó que estos dos métodos fueron los que presentaron menor capacidad para diferenciar especies del género *Candida* y también presentaron menores valores de Kp, en especial, cuando se comparó el método Vitek® 2 Compact con las otras metodologías evaluadas. Otros autores han reportado que estos métodos pueden presentar discrepancias para identificar especies del género *Candida* menos comunes y aquellas especies estrechamente relacionadas ^23^^,^^25^. Por otra parte, la exactitud de estos métodos se reduce en gran medida si los laboratorios no realizan las pruebas adicionales requeridas para la identificación de perfiles fenotípicos que ayuden a discernir entre las especies con discriminaciones bajas o que presenten dificultades en la identificación.

En nuestro estudio se identificaron por PCR panfungal y secuenciación, especies como *Candida fermentati* y *Candida haemulonii,* que son especies emergentes, clasificadas recientemente dentro de complejos de especies que tienen dificultad de identificarse por los métodos convencionales y cuya identificación se puede convertir en un gran desafío cuando estas cepas no han sido incluidas en las bases de datos de los diferentes sistemas de identificación ^26^^,^^27^. Además, algunas de estas especies emergentes han mostrado una disminución de la sensibilidad a los agentes antifúngicos, y en consecuencia, esto aumenta la dificultad en el tratamiento de los pacientes con infecciones invasivas por *Candida*. ^26^^,^^28^. En nuestro trabajo, ninguna de las metodologías analizadas, con excepción de la PCR panfungal y secuenciación, tuvo la capacidad de diferenciar las especies de *Candida lipolytica* (n= 1), *Candida fermentati* (n= 1) y *Pichia kluyveri* (n= 1), especies que si bien han sido reportadas principalmente para el uso a nivel industrial, también tienen la capacidad de comportarse como verdaderos patógenos en humanos de acuerdo con las condiciones del hospedero ^26^^,^^29^^-^^32^. 

Las metodologías basadas en MALDI TOF (Microflex® y Vitek® MS) presentaron una alta concordancia en comparación con el método de referencia, PCR panfungal y secuenciación. El análisis de concordancia entre estas dos tecnologías mostraron una concordancia muy buena, debido a que ambos sistemas fueron capaces de identificar el mismo número de especies pero no todas correspondieron a las mismas especies, ya que se obtuvieron incongruencias en las identificaciones en 6 de los 67 aislamientos analizados. Existen varias plataformas de MALDI TOF, las cuales presentan diferencias que van a depender de las bibliotecas de espectros de masas y de los algoritmos de identificación que tenga cada casa comercial ^33^^,^^34^. En nuestro estudio el Microflex® fue capaz de identificar todos los aislamientos a nivel de especie. Cuando se empleó el espectómetro Vitek® MS, se observaron tres discordancias mayores (esta plataforma no fue capaz de determinar la identificación final a nivel de especie en tres aislamientos), cuando se comparó con el método de referencia, lo cual afecto su valor de Kp. Muchos estudios han permitido determinar que estas técnicas son más rápidas y más precisas en la identificación de las diferentes levaduras a partir de cultivos, lo que tiene una alta relevancia a nivel de tiempo y costos de procesamiento ^7^^,^^35^^,^^36^. 

La ventaja de la PCR panfungal y secuenciación radica en que esta técnica permite identificar especies crípticas o complejos de especies, pero se recomienda combinar la amplificación de la región D1/D2 con otros blancos en la región ITS ^24^^-^^26^. En este trabajo, aunque no se observó la presencia de especies crípticas de *Candida parapsilosis* y *Candida glabrata*, es importante tener en cuenta la importancia de emplear metodologías que sean capaces de detectarlas, debido principalmente a la emergencia de especies con perfiles de sensibilidad de difícil manejo ^9^^,^^37^^,^^38^. Además, sería la mejor opción, a pesar de su costo, para aquellas identificaciones que por otras metodologías generen identificaciones dudosas o con bajas discriminaciones. 

Al realizar el análisis referente a los costos y al tiempo para la generación de resultados a nivel local, se pudo determinar que los métodos basados en la tecnología MALDI TOF (Microflex y Vitek® MS) fueron los que ofrecieron menores tiempos para la identificación de levaduras, como ha sido reportado en estudios previos, y al comparar esta metodología con los métodos convencionales, nuestros resultados reflejaron un costo muy razonable y competitivo en nuestro medio comparado con las demás técnicas ^39^. Cabe resaltar que esta tecnología permite resultados precisos, superando a las técnicas de identificación convencionales ^40^^,^^41^. Esta tecnología ha demostrado un gran potencial para una identificación rápida y precisa, y este hallazgo demuestra la necesidad de hacer que sus metodologías estén más disponibles.

Es importante mencionar que pueden ocurrir retrasos en la generación de resultados, debido a factores que no son inherentes al proceso, sino que dependen en gran parte de factores externos, como el día que llegue el aislamiento para su proceso, la viabilidad de los aislamientos, el requerimiento o no de un paso adicional de extracción, los días designados para el procesamiento de muestras, la disponibilidad de personal del laboratorio o el tiempo de respuesta para el análisis de las secuencias. Cambios en algunos de estos factores y conductas pueden marcar una gran diferencia a la hora de la entrega de un resultado oportuno y ayudarían a generar cambios a nivel de los laboratorios para mejorar los diferentes procesos.

El presente estudio se limitó a evaluar especies de *Candida* aisladas de mucosa oral y aunque se pudo evidenciar una gran variedad de especies identificadas entre los diferentes métodos, sería pertinente para futuras investigaciones realizar la misma evaluación de métodos en otras muestras clínicas. Adicionalmente, el análisis de costos y tiempos solo fue realizado en la ciudad de Medellín, Colombia. En Colombia, es limitado el número de laboratorio que ofrecen como servicio diagnóstico métodos moleculares y por tecnología MALDI TOF, lo cual puede incrementar de forma significativa los costos de estas pruebas, debido principalmente a la exclusividad del servicio.

## Conclusión

Es necesario implementar métodos de identificación que faciliten el acceso a resultados rápidos y confiables, pero que, a su vez ayuden a optimizar el recurso económico una vez sean implementadas en la rutina diaria. En este trabajo se evidenció que los sistemas basados en tecnología MALDI TOF, a pesar de ser metodologías que inicialmente requerían una inversión económica sustancial, demostraron ser rápidos, económicos (valor comercial de la prueba) y precisos, que se presentan como alternativas prometedoras para la identificación rutinaria de levaduras del género *Candida*. Para aquellos laboratorios que no pueden acceder a pruebas moleculares o de espectrometría de masas, el uso de pruebas automatizadas podrían ser una buena alternativa si el analista del laboratorio sigue las especificaciones proporcionadas por el fabricante y presta especial atención a los resultados que involucran especies poco comunes o características discrepantes en el aislamiento (morfología, patrón de susceptibilidad y otras claves taxonómicas adicionales).
